# Opportunities and Significance of Nanoparticle–DNA Binding in Medical Biotechnology: A Review

**DOI:** 10.7759/cureus.31005

**Published:** 2022-11-02

**Authors:** Harsh Verma, Muskan Aggarwal, Sunil Kumar

**Affiliations:** 1 Department of Medicine, Jawaharlal Nehru Medical College, Datta Meghe Institute of Medical Sciences, Wardha, IND

**Keywords:** dna structure, dna binding, genotoxicity, cytotoxicity, nanoparticles

## Abstract

Bionanotechnology is a budding area that combines engineering and biomedical sciences through the development of new functional systems and devices, especially related to medical use and their applications. DNA nanotechnology is one of the most widely studied fields for highly selective biosensing, imaging, drug delivery, and diagnostic purposes. Nanotechnology efficiently serves as a bridge between biotechnology and medical technology, and it offers great potential for medical and healthcare improvements. This has aroused a lot of scientific importance in the last few years owing to their exceptional practical capabilities and growing implementations in industrial and healthcare mechanics. Now, various research is being conducted to understand how nanoparticles can alter DNA sequences and their structures.

Recent experiments using circular dichroism and fluorescence tests show that nanoparticle binding produces a flexible conformational shift in the structure of DNA. Additionally, the nanoparticle's affinity for the DNA may very well be influenced by external agents, albeit the complex is stable at rather high ionic firmness.

This review gives an account of the mechanism of DNA-nanoparticle binding, with the binding of distinct types of nanoparticles and their interactions, how they change the structure of DNA, and their therapeutic uses and talks about their cytotoxic effects on humans and the environment.

## Introduction and background

Bionanotechnology is a brand new collaborative area of nanotechnology research and development (R&D) with biological, biochemical, or medicinal uses. To build unique devices such as biosensors, nanomedicines, and biophotonics, which are all made using biomimetic nanofabrication techniques, it mixes chemistry, biology, and molecular engineering; physical sciences; engineering; and biotechnology [1]. Many technologies based on nanoparticles (NPs) have proven to satisfy the need for biological material analysis in clinical diagnostics. NPs successfully bridge the gap between biotechnology and the medical tech field, and they hold a significant future for advancements in medicines and healthcare [1].

Due to their exceptional functional capabilities and growing uses in industrial and biomedical technologies, NPs have sparked an expanding number of studies in recent years. The evolution of molecular diagnostics has been aided by recent advances in NP technology, which provide considerable benefits over traditional diagnostic systems in terms of practicality, selectivity, and sensitivity in bioassays for DNA and protein markers [2]. NPs are additionally being considered as a potential transporter of DNA in target carriage to avoid the drawbacks of liposomes, which include limited encapsulation, fast blood leakage, and poor reserve stability. Using NPs to transmit plasmid DNA, small interfering RNA (siRNA), or antisense oligonucleotides can help avoid the unpredictability of viral vectors' immunological responses. As a result, NPs are projected to play an increasingly beneficial part in health sciences and technology in the coming years. The advancement of NP-based technology in healthcare and healthcare-related biotechnology is still a noteworthy study in the field of healthcare and its application in medicine, and thus, it will continue to encourage new developments in this area [1,2].

Several cutting-edge technologies based on NP-DNA binding and interactions have evolved and been applied in sensing, gene therapy, and molecular diagnosis. Gene regulation as a technique of managing clinical manifestations or changing cell function has emerged as a viable objective in medicinal chemistry [3]. When employing tiny particles or polymers, difficulties arise from building a suitable preorganized framework for regulated DNA recognition. The study of synthetic compounds capable of binding to DNA has led to the development of numerous kinds of engineered frameworks. In addition to systems that can covalently modify the target DNA strand, other approaches to the exploration for molecules suitable for interacting with DNA sequences have led to the discovery of peptide and saccharide scaffolds that use well-known DNA binding elements to give action to particular sequences. These methods offer a chance for the creation of effective and affordable DNA detection and high-sensitivity disease diagnosis systems [1,3].

## Review

This paper focuses on the principle of NP-DNA binding and the related interaction process. We exclusively concentrate on the most widely used gold (Au), silver (Ag), and carbon NPs and emphasize how they might be employed in molecular identification in medical biotechnology. Here, we discuss recent findings and ongoing research on how DNA molecular structure and bioactivities may change as a result of NP-DNA interaction. We briefly examine the potential effects of NPs on the toxicity of bacteria that have been exposed to them. The recent fusion of these interdisciplinary domains has given rise to the new study area of bionanotechnology, which is anticipated to have an increasingly significant future influence on the advancement of cutting-edge technologies. Recent studies have shown an increased interest in the molecular interactions and biochemical processes that occur between DNA and NPs.

Mechanism of DNA-NP binding

An NP-based approach is being developed for sensitive and specific diagnostics, as well as efficient gene delivery devices. One-to-one interactivities between a single DNA molecule and NPs via selective or nonspecific molecular binding can achieve high sensitivity. Anchor groups such as -COOH, -NH_2_, -OH, and -SH can be used to covalently attach DNA to the surface of NPs. The thiolated oligonucleotides are commonly functional with Au NPs or Ag NPs, resulting in DNA NP probes for their precise hybridization and identification of complementary sequences of interest [1]. Simple adsorption via noncovalent interactivities can achieve nonspecific binding between DNA and NPs. To regulate or limit the liberation of nucleic acids in gene therapy, the noncovalent binding affinity that is comparable to the interactions between repressor protein and DNA in vivo is needed. Therefore, to understand the architectural and practical basis for the fundamental mechanisms, knowledge of these molecular binding interactions at the atomic level is required. Short single-stranded DNA (24-mer ss-DNA) binds to 13 nm Au NP and avoids salt-induced aggregation [2]. Complementary hybridized oligomers, on the other hand, fail to stabilize unmodified Au NP and cause particle aggregation in a saline solution. These molecular binding processes are thought to be beneficial for testing mutations in the DNA and single nucleotide polymorphism (SNP) [1,2].

Binding of DNA with Au NPs

The selectivity of Au NPs for ss-DNA binding could be due to a variety of factors. Electrostatic interactions between anionic DNA strands and negatively charged surfaces of citrate-stabilized Au NPs, according to many researchers, make double-stranded DNA (ds-DNA) binding less favorable. The repulsion of ds-DNA with a higher surface charge density is greater than that of ss-DNA [1,3]. Furthermore, the research on deoxynucleoside binding affinity to Au NPs indicated that the four deoxynucleosides had significant bonds, although thymine links with the Au surface are considerably more fragile than the other nucleic bases [1,4]. The duplex DNA structure prevents the bases from coming into contact with Au surfaces, limiting DNA-Au-NP interactions [5]. Finally, ss-DNA is flexible and favors wrapping around Au NPs, while ds-DNA is relatively strong and not favorable for wrapping around the Au NPs [1,3].

As the length of the ss-DNA molecule grows longer, this binding weakens and happens only when the temperature increases, such as 55 °C for 50-mer ss-DNA. Hairpin structures are produced with relative ease in longer ss-DNA at room temperature, which can sterically inhibit binding interactions. Lengthy ss-DNA from 100-mer oligomers did not bind to 5 nm Au NPs [6]. Nonspecific ds-DNA (12 bp) binding to 13 nm Au NPs through ion-induced dipole dispersive interactivities was reported [7]. It was claimed that a conducting sphere's polarizability is proportional to the cube of the radius. When particle sizes vary, polarizability is shown to be dramatically diminished. One of the essential components in understanding the binding mechanism is the size influence of NPs. The affinity of ss-DNA binding to Au NPs was investigated and found that the strength of the interaction between ss-DNA and the surface of the Au particle is proportional to the particle size [8].

Binding of DNA with Ag NPs

Ample research has been carried out to produce simple and ultrasensitive Ag-NP-based devices for detecting specific DNA sequences [9]. Oligonucleotide-Ag-NP conjugates provided ultrasensitive DNA detection. It was found that the interactions between unmodified Ag NP and DNA molecules were sequence-dependent, implying that Ag NP and nucleobases have a close affinity [10]. In the UV-vis spectrum, Ag NP solutions containing the nucleic bases cytosine (C), guanine (G), adenine (A), and thymine (T) show redshift curves. The degree of nucleobase interaction varies in the order T < A < G < C, according to surface-enhanced Raman spectroscopy (SERS) intensity [10]. Atomic force microscopy (AFM) observation revealed interactions between Ag NP and genomic ds-DNA [11]. When isolated bacterial genomic DNA was treated with Ag NP (5 nm) for one hour, it was discovered that the NPs were randomly aligned to the ds-DNA. When long ss-DNA was incubated with Ag NP for one hour after thermal denaturation (95 °C for a couple of minutes and chilled immediately on ice) of genomic DNA, it showed efficient linking. The nonspecific binding of Ag NP to ds-DNA and ss-DNA was revealed by microscopic pictures [11]. When Ag NP was exposed to both genomic ss-DNA and ds-DNA, UV-vis spectra revealed a redshift. The zeta potential of Ag NP is negative. Thus, electrostatic contact is unlikely to result in binding to the polyanionic backbone's negatively charged phosphate groups. Coordination coupling involving two-dimensional (2D) orbitals, according to researchers, contributes to the development of Ag-NP-DNA complexes [1,11,12].

Binding of DNA with carbon nanomaterials and NPs

The links of DNA with carbon nanomaterials such as carbon nanotubes (CNTs), spherical fullerene, and carbon NPs also including CNT have been studied in a variety of experiments and theoretical models [13-16]. The helical structure of ss-DNA molecules was revealed to be bound to bundled single-walled CNTs (SWCNTs) [17,18]. ss- DNA molecules can be introduced within CNTs. Bundled SWCNTs were successfully diffused in water in the presence of ss-DNA and separated into a few fractions with various electronic architectures using ion exchange chromatography [19]. A hollow polygon consisting of 60 carbon atoms joined together in a highly symmetrical architecture is known as buckminsterfullerene (C60). C60 is of importance for scientific study and industrial uses due to its distinct physical and chemical features. C60 binds strongly to nucleotides in an aqueous solution through simulations. C60 attaches to ds-DNA at the hydrophobic ends of the nucleotide or in the slight groove. The nucleotides can be severely deformed by C60-ss-DNA interaction [15,16].

Scientists can use carbon NPs as nonviral transporters to carry DNA and RNA molecules into cells for gene therapy purposes if they understand how DNA and carbon NPs interact. Simulations of SWCNTs wrapped in ss-DNA revealed that the genome binds to CNT via pi-stacking interactions [19]. According to standard molecular dynamics simulations, the molecule wrapping around SWCNT may be driven by electrostatic and torsional interactivities inside the sugar-phosphate backbone [20]. The water-soluble C60 derivative ss-DNA binding process was discovered to be identical to native C60 DNA while creating more stable similar complexes [21]. The size and species of the functional groups determine how C60 derivatives attach to ss-DNA molecules. Between the ssDNA base rings and the benzene aromatic rings, there was a strong stacking. The hydrophilic contact between the chain and the ss-DNA backbone improves the DNA-C60 binding by a long hydrophilic chain. The hydroxyl hydrogen on the functional chain and the phosphate oxygen on the ss-DNA backbone generate stable hydrogen bonds. The hydrophobic contact between the C60 surfaces and nucleotide bases, as well as the many weak hydrogen bonds between the carboxyl or hydroxyl and the DNA backbone, dominate the binding of C60 derivatives with short hydrophilic groups [1].

NP-DNA binding in medical biotechnology

New NP-based techniques for faster and more reliable diagnosis of a sickness, cancer, and bioterrorism using DNA markers have been created. The signals from Raman scattering can be enhanced by Au NP by order of 105 to 1,015, allowing single molecule detection [22]. The use of Au NPs increased the sensitivity of surface plasmon resonance (SPR) by 1,000 times [23]. Updated research into NP-based DNA binding approaches will lead to well-established tests for several medical uses, taking advantage of distinctive size and optical features. Engineered Ag NPs are commonly employed to detect antibacterial/antiviral and superior catalytic properties, as well as to improve SERS [24]. The Ag unique NP's binding ability and features make it appealing for usage in medical diagnostics. The use of oligonucleotide-modified NPs will be able to improve DNA detection and transport efficiency through sequence-specific identification. However, covalent NP DNA binding makes its liberation for target medication challenges. Noncovalent binding appears to be essential for efficient DNA and small interfering RNA transport [1]. These NPs are typically covalently linked to either ss-DNA or an aptamer. The binding of NPs can result in hybridization or aptamer-target interactions, which cross-link the DNA molecules and NPs. The salts can significantly boost NP-induced DNA aggregation. For the detection of the DNA, unmodified NPs were also utilized [1]. Specific DNA sequencing, mutations, and SNPs can all benefit from NP-DNA binding. The hybridization of testing oligonucleotides may emerge in the formation of a concentration of NPs, which correlates to a color change of the solution, as NPs bind to ss-DNA. When the sample is heated, a single base mismatch causes the Tm profile to change. The advancement of existing assays allows for the quantitative detection of DNA at extremely low quantities. In clinical samples of genomic DNA, it is demonstrated that the use of the rapid technique to screen for SNPs is associated with a deadly arrhythmia known as long QT syndrome [2].

Gene Therapy

Nanostructured carbon materials have been applied frequently as scaffolds or vectors for medication transport. A diversity of hybridized composites of carbon NPs nucleic acids have been created to build nanoelectrical devices by gluing carbon nanostructures with particular sequence binding to the substrate [25]. Covalent bond formation or simple adsorption via noncovalent interactions can both be used to functionalize carbon nanostructures [26]. The combination of DNA technology and carbon nanomaterials has resulted in biomedical breakthroughs. For gene transport and DNA transfection, functional DNA-C devices and systems have been developed. Although DNA can be covalently coupled with functionalized carbon NPs, noncovalently attached composites have been made by wrapping CNT in DNA or inserting it into the NPs of concern and generating DNA/fullerene conjugates [27-29]. These innovative nonviral carriers have been shown to improve transfection efficiency and hold tremendous promise for future gene therapy applications [30].

Uptake, Cytotoxicity, and Genotoxicity of NPs

Human cells can take up NPs by endocytosis. Other pathways may potentially contribute to NP internalization according to studies conducted under endocytosis-inhibiting circumstances. NPs can be taken up by prokaryotic cells via phagocytosis due to a lack of endocytosis machinery. NPs can reach the nucleus after penetrating cellular membranes and passing through several cellular barriers. NPs attaching to DNA might thus cause long-term or chronic alterations, which would be difficult to detect in the short term but could be investigated further in the future [1].

The surface modification of NPs plays a crucial role in the cytotoxicity of Au NPs according to extensive cytotoxicity studies. A study investigated the intracellular utilization of unmodified NPs and concluded that cellular uptake is influenced by size and shape [31]. Au NPs could not cause acute cytotoxicity after entering the cells [32]. Taking account of findings of research conducted in 2004, cationic Au particles were somewhat hazardous, while anionic Au particles resulted to be harmless [33]. Polyethene glycol modification and protein adsorption onto Au NP result in a significant decrease in cellular absorption, lowering cytotoxicity in prostate cancer cells. In the past years, various exploration and analysis studies conducted on the immunogenic effects of Au NPs on macrophage cells have led to the findings that internalization of Au NPs within DNA did not stimulate the liberation of proinflammatory cytokines, tumor necrosis factor-alpha, and interleukin 1-beta, thus making them excellent candidates for nanomedicine [34].

Ag NPs have long been employed in wound dressings, catheters, and a variety of other goods due to their antibacterial properties. Because these NPs can quickly enter cells, there has recently been concern about their toxicity. Experiments in mice showed that Ag NPs caused inflammation and a change in lung function [35], while in vitro data showed apoptosis and DNA damage [36]. Ag NPs can be found in endosomes around cell membranes, throughout the cytoplasm, inside mitochondria, lysosomes, and the nucleus. The results of the experiments showed that Ag NPs were taken up via the endocytic pathway and were involved in mitochondrial toxicity and DNA damage. Ag NPs were also observed to lower adenine triphosphate (ATP) levels, implying that those NPs are involved in mitochondrial respiratory chain disruption and ATP synthesis interruption [37].

C60 was seen to accumulate intracellularly in lysosomes and cytoplasm, along the nuclear membrane, inside the nucleus, as well as along the plasma membrane. In macrophage cell lines, there was no evidence of pristine C60 toxicity [38]. C60, on the other hand, was discovered to have dose-dependent cytotoxicity in human dermal fibroblast and liver cancer cell lines [39]. Different cell lines responded differently to water-soluble fullerene derivatives. In Chinese hamster lung and Chinese hamster ovary (CHO) cells, hydroxylated C60 (Fullerenol) caused significant toxicity but had essentially little effect on L929 cells [40]. Fullerenol cytotoxicity was also discovered in human umbilical vein endothelial cells. In PC12 cells, the amino acid C60 derivatives can prevent oxidative stress-induced cell death without being harmful in HeLa cells, malonic acid C60 was reported to be cytotoxic or photocytotoxic [41,42]. Human dermal fibroblasts, human liver cancer cells (HepG2), and human neuronal astrocytes were found to be cytotoxic to aqueous C60 (nano-C60) [43].

## Conclusions

NPs have promising applications in cancer therapy, medical diagnosis, and genetics research, among other things, due to their chemical, optical, and electromagnetic capabilities. The optical and electrical sensitivities of Au and Ag NPs, as well as their many aggregates, allow them to be detected. In recent decades, selective detection of DNA linked with NPs has been achieved. The evolvement of novel technologies to distinguish a perfectly complementary target strand from a strand with a single base mismatch is enabled by DNA melting, hybridization, and the resulting color shift caused by DNA-Au-NP conjugating. Unmodified Au NPs bind to nucleotide bases, i.e., ss- and ds-oligonucleotides in distinct ways. SNPs have great potential when NPs bind to DNA. Many interesting ideas revolve around adsorbing Au/Ag NPs onto a DNA molecule to create conductive nanowires.

To manage disease states or change cellular activity, gene therapy, or regulation requires DNA binding affinity similar to protein-DNA interactions in vivo. As synthetic devices and assemblies capable of DNA binding, a variety of NPs and nanorods have been created. When it comes to creating DNA delivery methods, NPs outperform tiny particles and polymers. Functionalized Au NP, Ag NP, and fullerenes serve as excellent scaffolds for DNA transfection and the development of DNA-regulating compounds. In terms of ecotoxicology, cytotoxicity, and genotoxicology, NP-binding activities also pose dangers to the environment and human health. The effects of carbon NPs binding on DNA structure distortion and aggregation, which impact DNA bioactivity, present challenges for the evolution of DNA-NP-based technologies for medicinal and therapeutic implications. However, under specific experimental settings, Au NPs are thought to be relatively safe with minimal cytotoxicity.

The principles of molecular interactions between NPs and DNA, as well as NP-DNA binding mechanisms, are presented in this paper. In biological research and medical biotechnology, Au, Ag, and carbon NPs are being discovered with their broadest range of uses. Understanding the molecular interactions and mechanisms of NP-DNA binding will spur the evolution of novel biotechnologies in the coming years, allowing us to overcome the limitations of traditional diagnostics and therapy. The usage of NPs raises issues about ecotoxicity and genotoxicity, including DNA function modification, metabolic variance, gene regulation, and even population shift due to functional changes induced by the nanoparticle binding with the DNA. However, developing a new universal platform for modern electrical, diagnostic, and therapeutic instruments in medicine based on NP-DNA binding is a worthwhile long-term goal.
